# Selective Disruption of Perineuronal Nets in Mice Lacking Crtl1 is Sufficient to Make Fear Memories Susceptible to Erasure

**DOI:** 10.1007/s12035-023-03314-x

**Published:** 2023-04-06

**Authors:** Andrea Poli, Aurelia Viglione, Raffaele Mazziotti, Valentino Totaro, Silvia Morea, Riccardo Melani, Davide Silingardi, Elena Putignano, Nicoletta Berardi, Tommaso Pizzorusso

**Affiliations:** 1 BIO@SNS Lab, Scuola Normale Superiore Via G, Moruzzi 1, 56124 Pisa, Italy; 2 Institute of Neuroscience, National Research Council, Via Moruzzi, 1, 56124 Pisa, Italy; 3 Neuroscience Institute, New York University Grossman School of Medicine, New York, NY 10016 USA; 4 Department of Neuroscience, Psychology, Drug Research, and Child Health NEUROFARBA, University of Florence, 50134 Florence, Italy

**Keywords:** Perineuronal nets (PNNs), Chondroitin sulfate proteoglycans (CSPGs), Fear conditioning, Fear extinction, Pupillometry

## Abstract

The ability to store, retrieve, and extinguish memories of adverse experiences is an essential skill for animals’ survival. The cellular and molecular factors that underlie such processes are only partially known. Using chondroitinase ABC treatment targeting chondroitin sulfate proteoglycans (CSPGs), previous studies showed that the maturation of the extracellular matrix makes fear memory resistant to deletion. Mice lacking the cartilage link protein Crtl1 (Crtl1-KO mice) display normal CSPG levels but impaired CSPG condensation in perineuronal nets (PNNs). Thus, we asked whether the presence of PNNs in the adult brain is responsible for the appearance of persistent fear memories by investigating fear extinction in Crtl1-KO mice. We found that mutant mice displayed fear memory erasure after an extinction protocol as revealed by analysis of freezing and pupil dynamics. Fear memory erasure did not depend on passive loss of retention; moreover, we demonstrated that, after extinction training, conditioned Crtl1-KO mice display no neural activation in the amygdala (Zif268 staining) in comparison to control animals. Taken together, our findings suggest that the aggregation of CSPGs into PNNs regulates the boundaries of the critical period for fear extinction.

**Supplementary Information:**

The online version contains supplementary material available at 10.1007/s12035-023-03314-x.

## Introduction

The ability to extinguish fear memories when threats are no longer present is critical for adaptive behavior. During fear conditioning, the repeated pairing of an initially neutral stimulus (conditioned stimulus; CS) with an aversive stimulus (unconditioned stimulus; US) induces a strong and persistent fear memory [1] that can be inhibited by repeated exposure to the CS in the absence of the US, a process called fear extinction [2]. There is compelling behavioral evidence that extinction training does not erase or reverse the original CS-US association but rather leads to the formation of a new inhibitory memory that competes with the initial fear memory for the control of behavior [3, 4]. Fear memory extinction in adult animals is not permanent but decays with time, a process known as spontaneous fear recovery [5]. Moreover, conditioned fear responses can be restored by presenting the US alone in the context in which extinction training occurred (reinstatement) [4, 6] or may re-emerge following a shift in context (renewal) [7, 8]. The extinction of conditioned fear memories in adults relies on a network of structures, such as the basolateral amygdala (BLA), the lateral amygdala (LA), and the ventromedial prefrontal cortex (vmPFC) [9–12]. Previous studies have provided numerous lines of evidence showing that extinction training of adult animals produces a new memory that inhibits the original fear memory stored in the lateral amygdala (LA) [13, 14]. In contrast, extinction seems to produce a permanent erasure of fear memory in juvenile animals, which do not exhibit reinstatement or context-dependent renewal of conditioned fear responses following an extinction protocol [15–17]. The transition from a fear memory that can be erased in juvenile mice to a persistent fear memory in the adult have been suggested to rely on the maturation of the circuits involved in conditioned fear extinction [7]. For example, removal of chondroitin sulfate proteoglycans (CSPGs) from the amygdala extracellular matrix (ECM) by enzymatic digestion allowed juvenile-like erasure of conditioned fear memories in adult animals [7]. CSPGs are diffusely present in the ECM of the adult brain [18] and condense around some cells forming perineuronal nets (PNNs) [19]. This process is triggered by neuronal production of the cartilage link protein Crtl1 (also known as HAPLN1), which is upregulated during development [20, 21]. The developmental condensation of CSPGs in PNNs of the visual cortex, rather than their sheer presence, play a crucial role in protecting adult visual cortical circuits from being modified by experience [20]. However, it is unknown whether the condensation of CSPGs in PNNs, taking place during development, is also involved in the transition from a conditioned fear memory that can be erased by extinction to a fear memory that is no more susceptible to erasure. Also unknown is the mechanism through which PNNs make adult amygdala circuits resilient to extinction effects, protecting fear memories from erasure.

Here, we exploited mice lacking the Crtl1 protein (Crtl1-KO) which have attenuated PNNs but unchanged overall levels of CSPGs [20], to investigate whether preventing the aggregation of CSPGs into PNNs is sufficient to induce fear memory susceptible to erasure, and assessing the associated pattern of activation. In particular, we assessed fear responses through freezing and pupil size, two well-established behavioral and physiological markers of fear memories. Freezing is a defensive response commonly used to evaluate associative fear memory in rodents, while pupil dilation has been widely used to objectively assess fear learning in humans [22–24] and provides valuable information about the role of arousal in modulating fear circuits [25]. We found that Crtl1-KO animals retain the juvenile feature of erasing a specific conditioned fear memory following a protocol of extinction. In particular, Crtl1-KO mice exhibited a stronger reduction in both pupillary and freezing response to the CS with respect to Crtl1-WT mice. This persistent reduction of fear in Crtl1-KO mice did not depend on passive loss of memory, since fear memories assessed 9 days after learning without intervening extinction protocol is comparable in Crtl1-KO and Crtl1 wild-type mice (Crtl1-WT). To assess the mechanisms through which PNN disruption leads to permanent erasure of a fear memory, we analyzed neuronal activation in the amygdala and in the infralimbic cortex (IL) at the end of the extinction protocol via immunostaining for Zif268. We found that, following extinction, there was no neural activation in the amygdala of conditioned Crtl1-KO mice in response to CS, in accordance with the erasure of the conditioned fear memory. On the contrary, Crtl1-WT mice showed a clear activation of these regions.

## Results

### Lack of Crtl1 Accelerates Extinction of Fear Memories in Adult Crtl1-KO Mice

**Fig. 1:**
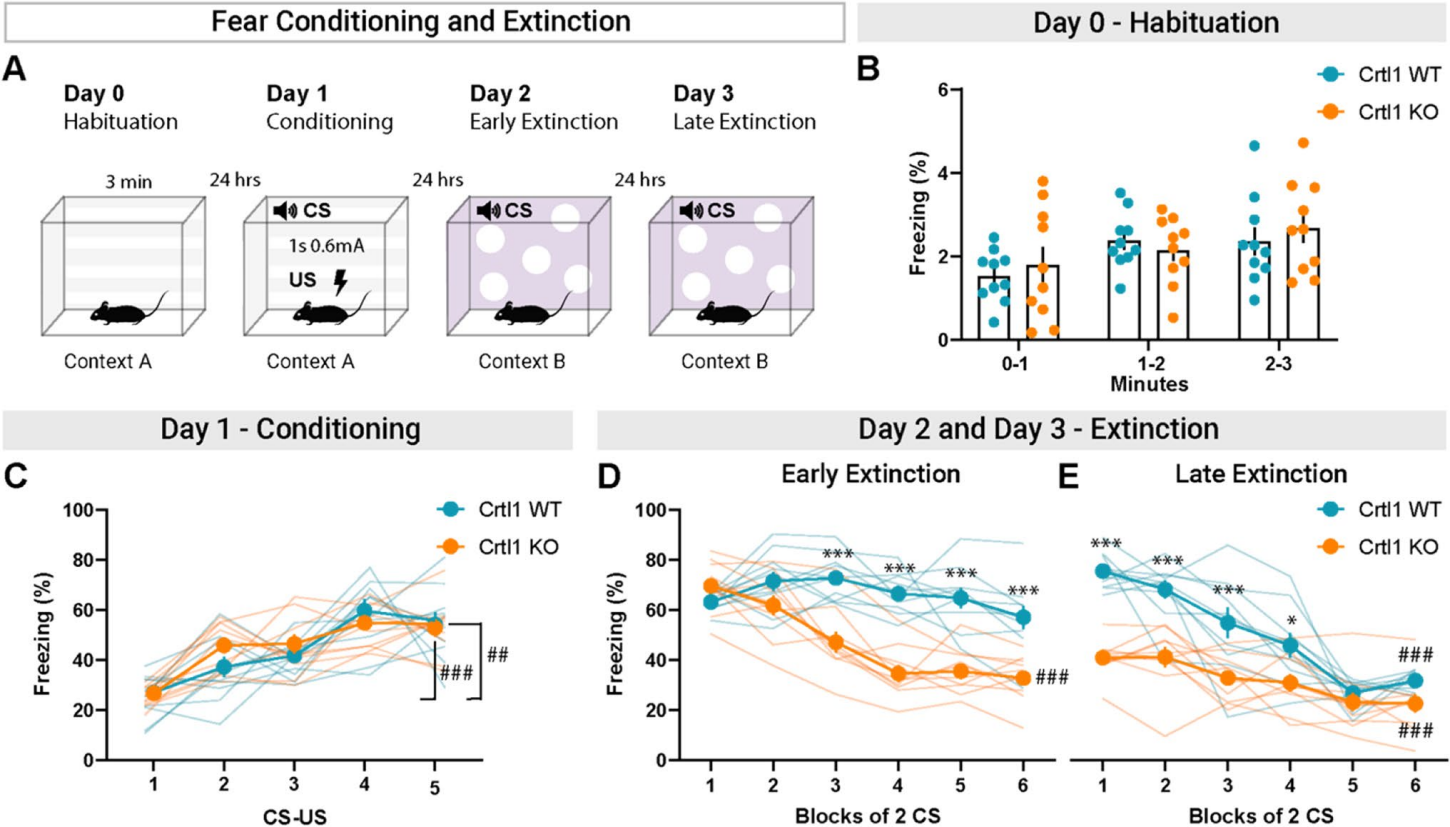
Fear extinction in Crtl1-KO mice. **A** Diagram showing the fear conditioning and extinction paradigm. **B** Habituation in Crtl1-KO and Crtl1-WT mice show low freezing levels and no differences between genotypes during 3-min exposition to the conditioned context. **C** Freezing levels in Crtl1-KO and Crtl1-WT mice during conditioning. Both genotypes exhibit a comparable pattern of freezing increase, coherent with a normal pattern of fear learning and no deficits of fear acquisition (two-way RM ANOVA: genotype *p* = 0.523, CS-US *p* < 0.001, interaction genotype x CS-US *p* = 0.346; post hoc Sidak multiple comparisons, CS-US within Crtl1-WT: 1 vs. 5 *p* < 0.01; CS-US within Crtl1-KO: 1 vs. 5 *p* < 0.001). **D** During early extinction, Crtl1-KO mice but not Crtl1-WT mice exhibited a significantly accelerated pattern of freezing reduction, as early as third block of 2 CS (two-way RM ANOVA: genotype *p* < 0.001; blocks of 2 CS *p* < 0.001; interaction genotype x blocks of 2 CS *p* < 0.001; post hoc Sidak multiple comparisons, genotype within blocks of 2 CS, 1: *p* = 0.721, 2: *p* = 0.251, 3: *p* < 0.001, 4: *p* < 0.001, 5: *p* < 0.001, 6: *p* < 0.001), and significantly reduced their freezing levels at the end of the extinction protocol (post hoc Sidak multiple comparisons, Crtl1-WT 1 vs. 6: *p* = 0.836; Crtl1-KO 1 vs. 6: *p* < 0.001). **E** During late extinction, Crtl1-KO mice kept showing significantly lower freezing from first to fourth block of 2 CS (two-way RM ANOVA: genotype *p* < 0.001, blocks of 2 CS *p* < 0.001, interaction genotype x blocks of 2 CS *p* < 0.001; post hoc Sidak multiple comparisons, genotype within blocks of 2 CS, 1: *p* < 0.001; 2: *p* < 0.001; 3: *p* < 0.001; 4: *p* < 0.05; 5: *p* = 0.980; 6: *p* = 0.369) and significantly reduced their freezing levels (post hoc Sidak multiple comparisons, Crtl1-KO 1 vs. 6: *p* < 0.001). From the fifth block of 2 CS, Crtl1-WT mice reached freezing levels comparable to Crtl1-KO and significantly reduced their freezing levels (post hoc Sidak multiple comparisons, Crtl1-WT 1 vs. 6: *p* < 0.001). *n* = 10 Crtl1-KO, *n* = 10 Crtl1-WT. *P-value between genotypes, #*P*-value between CS. CS = conditioned stimulus; US = unconditioned stimulus

To investigate whether the condensation of CSPGs in PNNs is crucial in the transition from a conditioned fear memory that can be erased by extinction to a conditioned fear memory which is not erasable, we performed a classical auditory cued fear conditioning and extinction protocol in Crtl1-KO mice and their WT littermates (Fig. 1A). Crtl1-KO mice exhibit a marked decrease in the amount of PNNs in the amygdala and IL cortex (Suppl. Figure 1), key regions for the extinction of adult conditioned fear memories. During the habituation, mice freely explored the chamber (day 0, context A) showing low levels of freezing (below 4%), consistently with a normal habituation to the context, with no difference between genotypes (Fig. 1B). We also found comparable freezing levels between conditioned Crtl1-KO and Crtl1-WT mice during the learning phase of the test (day 1, Fig. 1C). However, we found that Crtl1-KO mice exhibited a significantly accelerated pattern of freezing reduction with respect to Crtl1-WT mice (Fig. 1D) during the first day of extinction (early extinction, day 2). In particular, freezing levels in Crtl1-KO become significantly lower than in Crtl1-WT mice as early as the third block of 2 conditioned stimuli (CS), reaching significantly lower levels of freezing with respect to the beginning of CS presentations (Fig. 1D) at the end of the first day of extinction. During the second day of the extinction protocol (late extinction, day 3), Crtl1-KO mice maintain the low freezing levels achieved during the first day of extinction (Fig. 1E), while Crtl1-WT mice began to decrease freezing, reaching levels comparable to Crtl1-KO mice from the fifth block of 2 CS (Fig. 1E). These results demonstrate that CSPG condensation in PNNs due to cartilage link protein Crtl1 has an important role in promoting an accelerated fear memory extinction but not in learning.

### Pupillometry as a Physiological Readout of Fear Extinction in Crtl1-KO Mice

**Fig. 2:**
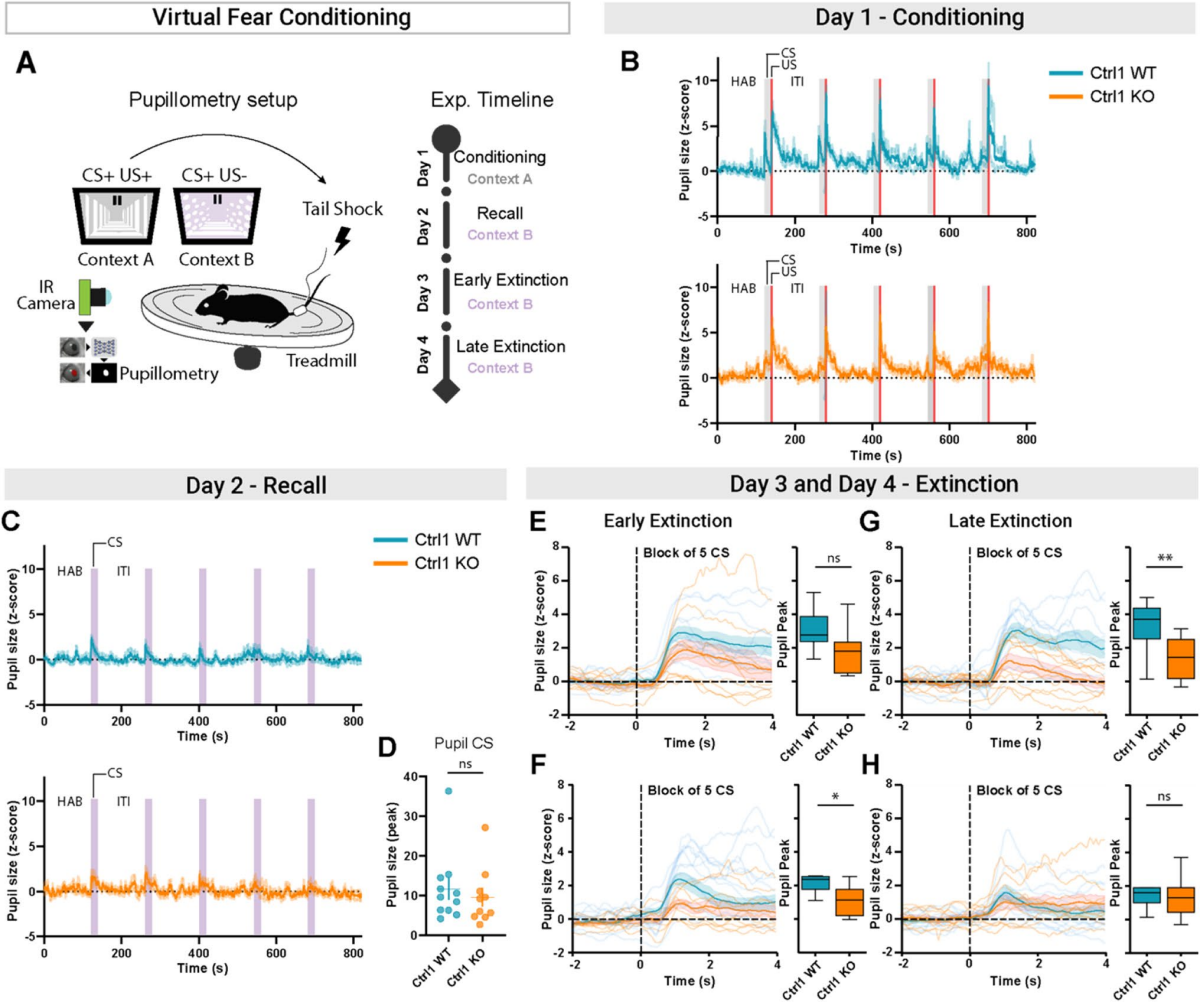
Pupillometry assessment of fear extinction in Crtl1-KO mice. **A** Diagram showing the pupillometry setup and the virtual fear conditioning timeline. **B** Average of the pupillary responses of Crtl1-WT (top) and Crtl1-KO (bottom) mice during the virtual fear conditioning. The gray area represents the presentation of the CS stimulus and the red area the presentation of the US stimulus. **C** Average of the pupillary responses of Crtl1-WT (top) and Crtl1-KO (bottom) mice during the virtual fear recall. The purple area represents the presentation of the CS stimulus. **D** During fear recall, we observed comparable pupillary responses between genotypes to the CS stimulus (unpaired *T*-test *p* = 0.566). **E** On the left, the average fluctuation of pupil size for the first block of 5 CS during early extinction. On the right, the pupil peaks during the presentation of the first 5 CS. We found no differences between genotypes (unpaired *T*-test *p* = 0.208). **F** On the left, the average fluctuation of pupil size for the second block of 5 CS during early extinction. On the right, the pupil peaks during the presentation of the second block of 5 CS. We found a lower pupillary response in Crtl1-KO mice compared to Crtl1-WT mice (unpaired *T*-test *p* = 0.010). **G** On the left, the average fluctuation of pupil size for the first block of 5 CS during late extinction. On the right, the pupil peaks during the presentation of the first 5 CS. We still found a lower pupillary response in Crtl1-KO mice compared to Crtl1-WT mice (unpaired *T*-test *p* < 0.01). **H** On the left, the average fluctuation of pupil size for the second block of 5 CS during late extinction. On the right, the pupil peaks during the presentation of the second 5 CS. We found no differences between genotypes (unpaired *T*-test *p* = 0.527). *n* = 10 Crtl1-KO, *n* = 11 Crtl1-WT. CS = conditioned stimulus; US = unconditioned stimulus; HAB = habituation; ITI = inter-trial; ns = not significant

Due to its sensitivity to arousal [25–27], the pupil responds with dilation to salient or threatening stimuli. For this reason, pupil dilations have gained interest as a measure of the conditioned response [23]. We used pupillometry as a physiological readout of fear learning and extinction in Crtl1-KO mice and their WT littermates. We designed a virtual cued fear conditioning protocol, using a visual cue as CS, paired with a tail shock (US). During fear conditioning, mice were head-fixed and free to run on a circular treadmill. An infrared webcam was used to record the pupil and the MEYE Deep Learning tool was employed to perform pupillometry [28] (Fig. 2A). To assess the efficacy of our virtual fear conditioning protocol, first, we tested C57BL6/J wild-type mice receiving the CS alone (sham) or the CS paired with the US (shock) (Suppl. Figure 2A). As shown in Suppl Fig. 2, we observed a stronger pupil dilations in response to CS in shocked mice compared to sham mice the day after conditioning (recall, Suppl. Figure 2C, D) validating the use of pupil size measurement to reveal learned fear. Then, we evaluated fear learning in Crtl1-KO and Crtl1-WT mice (Fig. 2) in a cohort of mice different from the one used for freezing assessment. As assessed with freezing levels, we observed comparable pupillary response between genotypes to the CS after learning (Fig. 2B-D). Interestingly, we found that Crtl1-KO mice exhibited a stronger reduction in the pupillary response to CS with respect to Crtl1-WT mice during the first day of extinction (early extinction) (Fig. 2 E, F). Pupillary responses in Crtl1-KO become significantly lower than in Crtl1-WT mice as early as the second block of 5 CS (Fig. 2F). During the second day of the extinction protocol (late extinction), Crtl1-KO mice still showed significantly lower pupillary responses compared to Crtl1-WT mice (Fig. 2G). Crtl1-WT mice reached pupillary responses comparable to Crtl1-KO mice from the second block of 5 CS of late extinction (Fig. 2H). To exclude possible defects in the pupillary light response present in mutant mice, we evaluated the pupillary light reflex (PLR). The results revealed in Crtl1-KO mice an unaltered PLR during both constriction and pupil re-dilation (Suppl. Figure 3).

Thus, we observed a faster extinction of fear memories in Crtl1-KO mice also using a physiological measure.

### Abnormal Spontaneous Recovery and Fear Renewal in Adult Crtl1-KO Mice

**Fig. 3:**
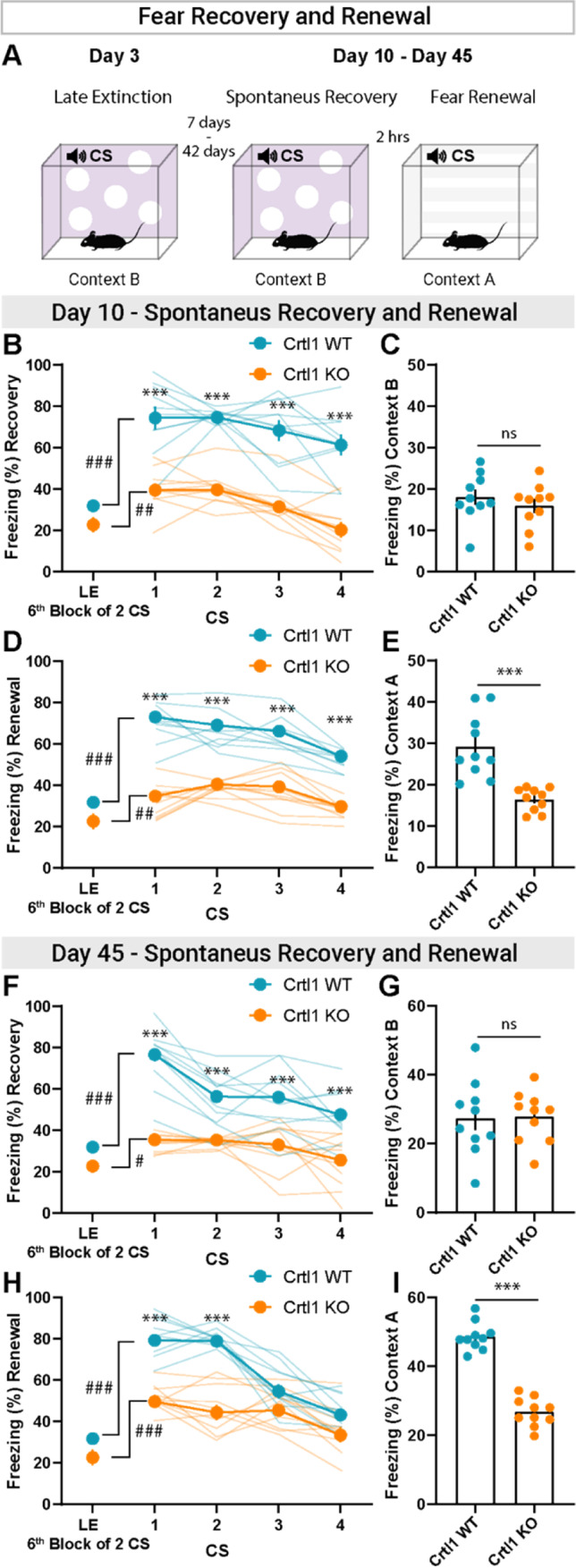
Spontaneous recovery and fear renewal in Crtl1-KO mice. **A** Diagram showing the spontaneous recovery and fear renewal paradigm. **B** Spontaneous recovery 7 days after extinction (day 10). Crtl1-KO mice kept showing significantly lower freezing during all four CS presentations with respect to Crtl1-WT mice (two-way RM ANOVA, interaction genotype x CS *p* < 0.001, genotype *p* < 0.001, CS *p* < 0.001; post hoc Sidak multiple comparisons, genotype within CS, 1: *p* < 0.001, 2: *p* < 0.001, 3: *p* < 0.001, 4: *p* < 0.001). **C** Crtl1-KO and Crtl1-WT mice showed the same freezing levels when placed in the unconditioned context B, 7 days after extinction (day 10) (unpaired *T*-test, *p* = 0.408). **D** Fear renewal 7 days after extinction (day 10). Crtl1-KO mice kept showing significantly lower freezing during all four CS presentations with respect to Crtl1-WT mice (two-way RM ANOVA, interaction genotype x CS *p* < 0.001, genotype *p* < 0.001, CS *p* < 0.001; post hoc Sidak multiple comparisons, genotype within CS, 1: *p* < 0.001, 2: *p* < 0.001, 3: *p* < 0.001, 4: *p* < 0.001). **E** Crtl1-WT mice showed a higher context-dependent freezing behavior compared to Crtl1-KO mice when placed in the conditioned context A, 7 days after extinction (day 10) (unpaired *T*-test, *p* < 0.001). **F** Spontaneous recovery 42 days after extinction (day 45). Crtl1-KO mice kept showing significantly lower freezing during all four CS presentations with respect to Crtl1-WT mice (two-way RM ANOVA, interaction genotype x CS *p* < 0.001, genotype *p* < 0.001, CS *p* < 0.001; post hoc Sidak multiple comparisons, genotype within CS, 1: *p* < 0.001, 2: *p* < 0.001, 3: *p* < 0.001, 4: *p* < 0.001). **G** Crtl1-KO and Crtl1-WT mice showed the same freezing levels when placed in the unconditioned context B, 42 days after extinction (day 45) (unpaired *T*-test, *p* = 0.928). **H** Fear renewal 42 days after extinction (day 45). Crtl1-KO mice kept showing significantly lower freezing during the first two CS presentations with respect to Crtl1-WT mice (two-way RM ANOVA, interaction genotype x CS *p* < 0.001, genotype *p* < 0.001, CS *p* < 0.001; post hoc Sidak multiple comparisons, genotype within CS, 1: *p* < 0.001, 2: *p* < 0.001, 3: *p* = 0.127, 4: *p* = 0.092). **I** Crtl1-WT mice showed a higher context-dependent freezing behavior compared to Crtl1-KO mice when placed in the conditioned context A, 42 days after extinction (day 45) (unpaired *T*-test, *p* < 0.001). *n* = 10 Crtl1-KO, *n* = 10 Crtl1-WT. *P-value between genotypes, #*P*-value between CS. CS = conditioned stimulus; ns = not significant; LE = late extinction

Seven days after late extinction (day 10), we assessed spontaneous recovery and fear renewal (Fig. 3A). The results clearly showed that Crtl1-KO mice still displayed attenuation of fear response caused by the extinction protocol while Crtl1-WT mice exhibited a higher fear response retrieval, both for spontaneous recovery (Fig. 3B) and for fear renewal (Fig. 3D). Moreover, the lower freezing level of Crtl1-KO mice remained evident throughout repetition of CS, both for spontaneous recovery and for fear renewal protocols. Interestingly, we also found that both Crtl1-KO and Crtl1-WT mice showed the same freezing levels when placed in the unconditioned context (context B, spontaneous recovery) (Fig. 3C), while Crtl1-WT mice showed a higher context-dependent freezing behavior compared to Crtl1-KO mice when placed in the conditioned context (fear renewal, context A) (Fig. 3E).

Assessment of spontaneous recovery and fear renewal 42 days after the end of the late extinction (day 45, Fig. 3A) showed that Crtl1-KO mice still displayed lower fear response caused by the extinction protocol compared to Crtl1-WT (Fig. 3F and Fig. 3H). The lower freezing level of Crtl1-KO mice with respect to Crtl1-WT mice during spontaneous recovery remained evident throughout repetition of CS (Fig. 3F). When the fear renewal protocol was employed, we found that Crtl1-KO mice showed significantly lower freezing during the 1 and 2 CS with respect to Crtl1-WT mice, while, during the 3 and 4 CS, Crtl1-WT mice reached freezing levels comparable to Crtl1-KO mice (Fig. 3H). Again we found that both genotypes showed comparable freezing levels in the unconditioned context (context B, spontaneous recovery) (Fig. 3G), while Crtl1-WT mice showed a higher context-dependent freezing behavior compared to Crtl1-KO mice when placed in the conditioned context (fear renewal, context A) (Fig. 3I).

Taken together, these results suggest that PNN disruption in Crtl1-KO mice is sufficient to determine a juvenile-like fear extinction: Crtl1-KO mice show a persistent reduction of fear both in spontaneous recovery and in context-dependent renewal, 7 and 42 days after extinction; Crtl1-WT mice show, instead, the lack of long-term effects of extinction typical of adults.

### Reduction of Fear in Crtl1-KO Mice Did Not Depend on Passive Loss of Memory

**Fig. 4:**
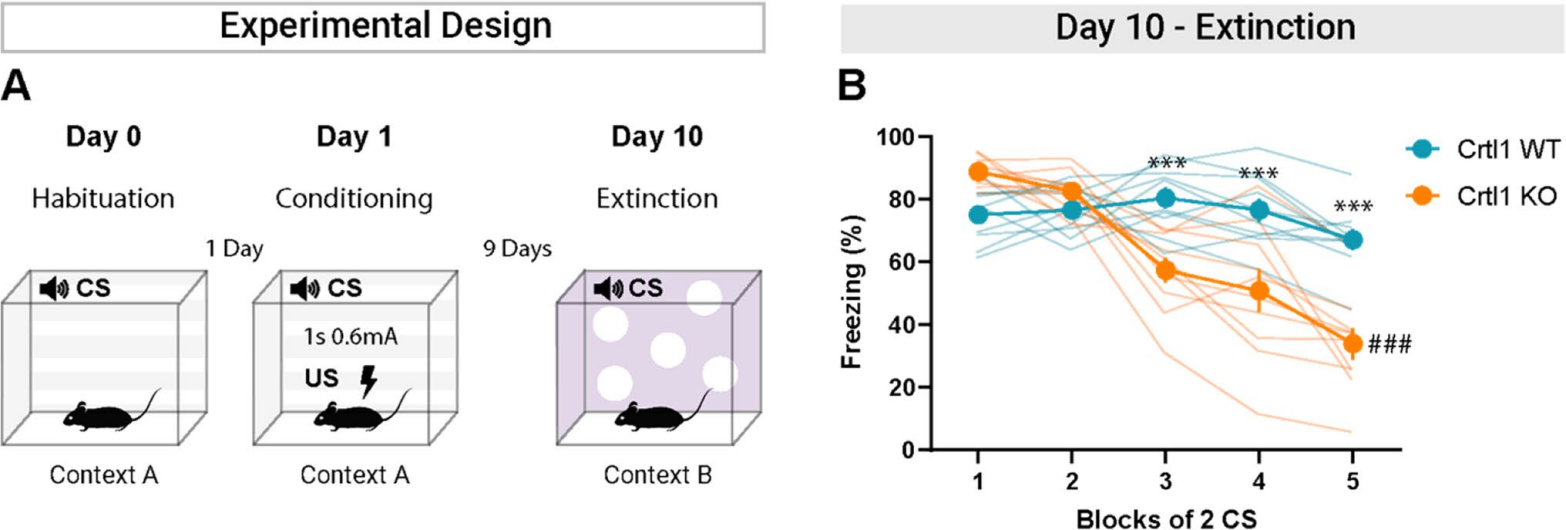
Freezing levels in conditioned Crtl1-KO and Crtl1-WT mice during early extinction starting 9 days after fear conditioning. **A** Diagram showing the fear conditioning and extinction paradigm. **B** Even if the extinction procedure started 9 days after fear learning, Crtl1-KO mice, but not Crtl1-WT mice, exhibited a significantly accelerated pattern of freezing reduction, as early as third block of 2 CS (two-way RM ANOVA: genotype, *p* < 0.01; blocks of 2 CS: *p* < 0.001; interaction genotype x blocks of 2 CS: *p* < 0.001; post hoc Sidak multiple comparisons, genotype within blocks of 2 CS, 1: *p* = 0.059, 2: *p* = 0.794, 3: *p* < 0.001, 4: *p* < 0.001, 5: *p* < 0.001), and significantly reduced their freezing levels during early extinction (post hoc Sidak multiple comparisons, blocks of 2 CS Crtl1-WT, 1 vs. 5: 0.520, Crtl1-KO, 1 vs. 5: *p* < 0.001). *n* = 10 Crtl1-KO, *n* = 10 Crtl1-WT. *P-value between genotypes, #*P*-value between CS. CS = conditioned stimulus; US = unconditioned stimulus

To ensure that the reduction in fear memory observed in Crtl1-KO mice after extinction was not due to a weakened consolidation and retention of the memory, we conducted a fear memory extinction test 9 days after fear conditioning (Fig. 4A). We found that freezing levels in the 1 and 2 blocks of 2 CS did not differ between Crtl1-WT and Crtl1-KO mice (Fig. 4B). This result confirms that Crtl1-KO mice do not show deficits in fear memory consolidation and retention. Moreover, we observed a faster decrease in fear response in Crtl1-KO mice compared to Crtl1-WT mice (Fig. 4B). Also in this case, Crtl1-KO, but not Crtl1-WT mice, significantly reduced their freezing levels at the end of the early extinction protocol (Fig. 4B). These results suggest that the higher efficacy of the extinction protocol in Crtl1-KO mice is present for both recent and older memories.

### Fear Extinction in Adult Crtl1-KO Mice is Accompanied by a Complete Loss of Amygdala Activation in Response to the CS

**Fig. 5:**
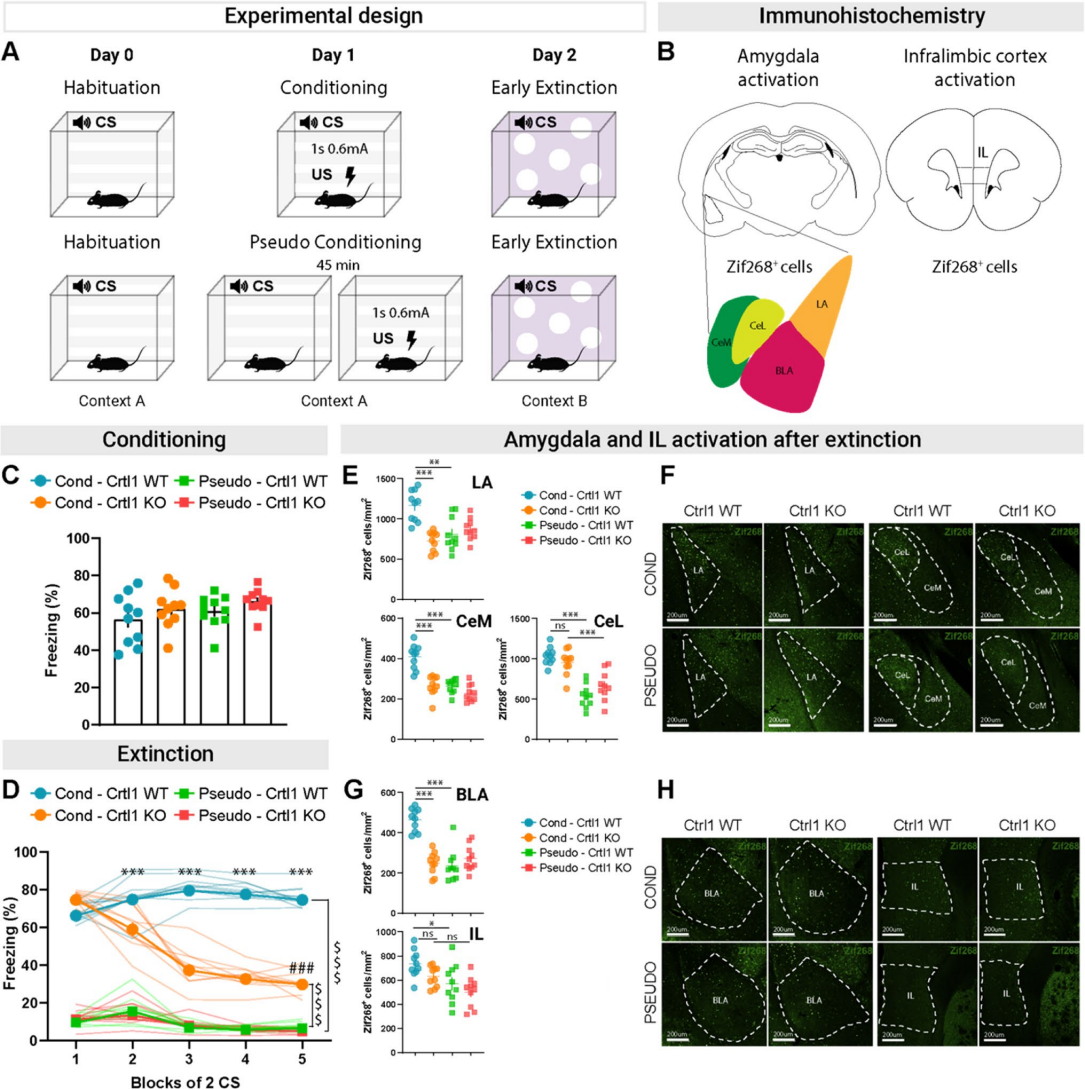
Amygdala and IL cortex activation after extinction in Crtl1-KO mice. **A** Diagram showing the experimental design. **B** Representative diagram of the areas considered for the Zif268 immunohistochemistry: BLA, basolateral amygdala; LA, lateral amigdala; CeM, medial part of the central amygdala; CeL, lateral part of the central amygdala; IL, infralimbic cortex. **C** Freezing levels in conditioned and pseudo-conditioned Crtl1-KO and Crtl1-WT mice at the end of the conditioning protocol. After 5 presentations of US, pseudo-conditioned Crtl1-KO and Crtl1-WT mice reached freezing levels comparable to conditioned mice (one-way ANOVA, *p* = 0.216). **D** Freezing levels in conditioned and pseudo-conditioned mice during the early extinction. Cond-Crtl1-KO mice exhibited a significantly accelerated pattern of freezing reduction (three-way ANOVA, genotype x condition x blocks of 2 CS *p* < 0.001. Genotype *p* < 0.001, condition: *p* < 0.001, blocks of 2 CS *p* < 0.001; genotype x condition *p* < 0.001, genotype x blocks of 2 CS: *p* < 0.001, condition x block of 2 CS, *p* < 0.001; post hoc Sidak multiple comparisons, difference between Cond-Crtl1-KO and Cond-Crtl1-WT, CS-1: *p* = 0.218, CS-2: *p* < 0.001, CS-3: *p* < 0.001, CS-4: *p* < 0.001, CS-5: *p* < 0.001) and significantly reduced their freezing levels during early extinction (post hoc Sidak multiple comparisons, difference between freezing levels for the 1 and 5 blocks of 2 CS Crtl1-WT, *p* = 0.200; Crtl1-KO, *p* < 0.001). Pseudo-Crtl1-WT and pseudo-Crtl1-KO mice did not show any significant difference of freezing levels between genotypes but significantly lower freezing levels during all five blocks of 2 CS with respect to conditioned mice (post hoc Sidak multiple comparisons, condition within blocks of 2 CS, Cond-Crtl1-WT, 1 to 5: *p* < 0.001; Cond-Crtl1-KO, 1 to 5: *p* < 0.001). **E** Analysis of Zif268-positive cells in the LA (top), CeM (bottom left), and CeL (bottom right) of conditioned and pseudo-conditioned mice. Cond-Crtl1-KO mice showed significantly reduced levels of LA and CeM activation with respect to Cond-Crtl1-WT mice (LA, two-way ANOVA: genotype *p* < 0.01; condition *p* = 0.051; interaction genotype x condition *p* < 0.001. Post hoc Sidak multiple comparisons: Cond-Crtl1-WT vs. Cond-Crtl1-KO *p* < 0.001, Cond-Crtl1-WT vs. pseudo-Crtl1-KO *p* < 0.01) (CeM, two-way ANOVA: genotype *p* < 0.001; condition *p* < 0.001; interaction genotype x condition *p* < 0.001. Post hoc Sidak multiple comparisons: Cond-Crtl1-WT vs. Cond-Crtl1-KO *p* < 0.001, Cond-Crtl1-WT vs. pseudo-Crtl1-KO *p* < 0.001) and indistinguishable from those shown by pseudo-Crtl1-WT and pseudo-Crtl1-KO mice (LA, post hoc Sidak multiple comparisons: Cond-Crtl1-KO vs. pseudo-Crtl1-WT *p* = 0.858, Cond-Crtl1-KO vs. pseudo-Crtl1-KO *p* = 0.349) (CeM, post hoc Sidak multiple comparisons: Cond-Crtl1-KO vs. pseudo-Crtl1-WT *p* > 0.99, Cond-Crtl1-KO vs. pseudo-Crtl1-KO *p* = 0.430). Conditioned mice showed higher levels of CeL activation compared to pseudo-conditioned mice (two-way ANOVA: genotype *p* = 0.630; condition *p* < 0.001; interaction genotype x condition *p* < 0.05. Post hoc Sidak multiple comparisons: condition within WT mice *p* < 0.001, condition within KO mice *p* < 0.001). **F** Representative images of Zif268-positive cells in LA, CeM, and CeL of conditioned and pseudo-conditioned Crtl1-WT and Crtl1-KO mice after the fifth block of 2 CS during early extinction. Scale bar, 200 μm. A larger version of this image is present in Suppl. Figure 5. **G** Analysis of Zif268-positive cells in the BLA (top) and IL (bottom) of conditioned and pseudo-conditioned Crtl1-WT and Crtl1-KO mice. Cond-Crtl1-KO mice showed significantly reduced levels of BLA activation with respect to Cond-Crtl1-WT mice (two-way ANOVA: genotype, *p* < 0.001; condition, *p* < 0.001; interaction genotype x condition, *p* < 0.001. Post hoc Sidak multiple comparisons: Cond-Crtl1-WT vs. Cond-Crtl1-KO *p* < 0.001, Cond-Crtl1-WT vs. pseudo-Crtl1-KO *p* < 0.001) and indistinguishable from those shown by pseudo-Crtl1-WT and pseudo-Crtl1-KO mice (post hoc Sidak multiple comparisons: Cond-Crtl1-KO vs. pseudo-Crtl1-WT *p* = 0.997, Cond-Crtl1-KO vs. pseudo-Crtl1-KO *p* = 0.965). Cond-Crtl1-KO mice showed comparable levels of IL activation such as that shown by Cond-Crtl1-WT mice (two-way ANOVA: genotype *p* < 0.05; condition *p* < 0.001; interaction genotype x condition *p* = 0.634. Post hoc Sidak multiple comparisons: genotype within conditioned mice *p* = 0.124, genotype within pseudo-conditioned mice *p* = 0.401), while pseudo-Crtl1-WT but not pseudo-Crtl1-KO mice showed reduced Zif268-positive cell density with respect to conditioned mice (post hoc Sidak multiple comparisons: condition within WT, *p* < 0.05; condition within KO, *p* = 0.058). **H** Representative images of Zif268-positive cells in BLA and IL of conditioned and pseudo-conditioned Crtl1-WT and Crtl1-KO mice after the fifth block of 2 CS during early extinction. Scale bar, 200 μm. A larger version of this image is present in Suppl. Figure 5. *n* = 10 in each group. *P-value between genotypes, #*P*-value between CS, $*P*-value between conditions. CS = conditioned stimulus; US = unconditioned stimulus

To assess neuronal activation in the amygdala and IL cortex of Crtl1-KO mice, we performed an immunostaining for Zif268. Zif268 is an immediate early gene known to be implicated in neuronal plasticity and memory formation [29]. We focused the analysis on the early extinction stage, corresponding to the maximal difference between Crtl1-KO and Crtl1-WT mice. To isolate the specific effect of associative learning on amygdala and IL activation from the effect of exposure to the CS and to the US, we compared conditioned mice to animals that received tone and electrical stimulation in an unpaired pattern (pseudo-conditioned mice) [30] (Fig. 5A).

The behavioral results show that both conditioned and pseudo-conditioned Crtl1-KO and Crtl1-WT mice showed a comparable response to the US, reaching a freezing level around 60% at the end of conditioning (Fig. 5C). However, conditioned mice developed a strong response to the CS (Fig. 5D). The results confirmed the enhanced extinction of Crtl1-KO mice by revealing a faster reduction of fear response in Crtl1-KO compared to Crtl1-WT mice (Fig. 5D). As expected, pseudo-conditioned mice showed significantly lower levels of freezing with respect to conditioned mice, and did not show any significant reduction of freezing levels with extinction (Fig. 5D). Therefore, this group of animals could be used to test Zif268 activation.

It has been shown that neurons in lateral amygdala (LA) maintain high levels of response to CS even after extinction protocols [31]. However, we found that there was no significant neuronal activation in the LA of conditioned Crtl1-KO mice after extinction, while neuronal activation was clearly present in conditioned Crtl1-WT mice compared to pseudo-conditioned Crtl1-WT mice (Fig. 5E). Density for Zif268 + cells in the main output nucleus of the amygdala, the medial part of the central amygdala (CeM), resulted significantly higher in conditioned Crtl1-WT mice than in pseudo-conditioned Crtl1-WT mice, showing significant neuronal activation in CeM (Fig. 5E). In conditioned Crtl1-KO mice, there was no significant difference in Zif268 + cell density with respect to Crtl1-KO pseudo-conditioned mice, showing absence of neuronal activation in CeM (Fig. 5E), in accordance with behavioral results of very low fear response at the end of early extinction (Fig. 5D). Neuronal activation in conditioned Crtl1-KO mice was significantly lower than in conditioned Crtl1-WT mice, while no difference in neuronal activation was found between the two pseudo-conditioned groups (Fig. 5E). We also found that density for Zif268 + cells in the lateral part of the central amygdala (CeL) resulted significantly higher in both conditioned Crtl1-WT and Crtl1-KO mice than in pseudo-conditioned mice, showing significant neuronal activation in CeL after extinction (Fig. 5E). Thus, the lack of CeM activation in Crtl1-KO mice seems to be due to lack of LA activation (Fig. 5E-F).

BLA is a crucial part of the intra-amygdala circuitry, providing a major point of control in the transmission of information between LA and CeM, and in particular is an important site of modulation of LA-CeM transmission during extinction [12, 32, 33]. Density of Zif268 + cells in the BLA showed the same pattern found in CeM, with only conditioned Crtl1-WT mice showing significant neuronal activation in response to CS (Fig. 5G). Thus, the absence of neuronal activation in Crtl1-KO mice is already present in BLA, one of the main inputs to CeM (Fig. 5G-H). We also assessed neuronal activation in IL during early extinction as it seems to play a relevant role in reducing fear response during extinction protocols [11, 34]. We found that both conditioned Crtl1-WT and Crtl1-KO mice showed significant IL activation at the end of early extinction although there was a strong trend for lower IL activation in KO with respect to WT mice (Fig. 5G-H). Intriguingly, Zif268 induction in conditioned Crtl1-WT mice was observed also restricting Zif268 analysis to cells positive for WFA (Suppl. Figure 4).

**Fig. 6:**
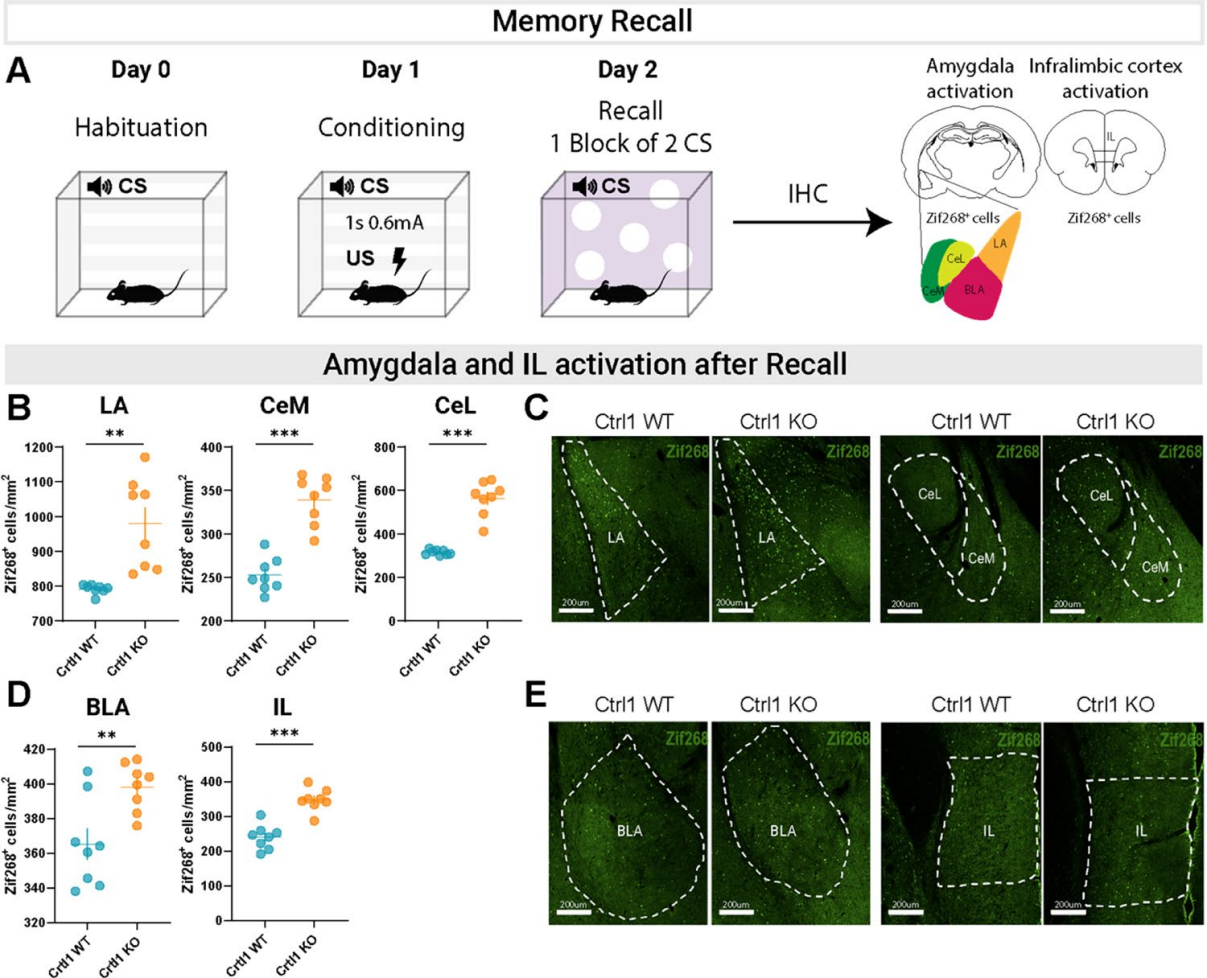
Amygdala and IL activation after memory recall in Crtl1-KO mice. **A** Diagram showing the experimental design. **B** Analysis of Zif268+ cells in the LA, CeM, and CeL of Crtl1-WT and Crtl1-KO mice. Crtl1-KO mice showed significantly higher levels of LA, CeM, and CeL activation with respect to Crtl1-WT mice (unpaired *T*-test, LA: *p* = 0.001, CeM: *p* < 0.0001, CeL: *p* < 0.0001). **C** Representative images of Zif268+ cells in LA, CeM, and CeL of Crtl1-WT and Crtl1-KO mice after memory recall. Scale bar, 200 μm. **D** Analysis of Zif268+ cells in the BLA and IL of Crtl1-WT and Crtl1-KO mice. Crtl1-KO mice showed significantly higher levels of BLA and IL activation with respect to Crtl1-WT mice (unpaired *T*-test, BLA: *p* = 0.006, IL: *p* < 0.0001). **E** Representative images of Zif268+ cells in BLA and IL of Crtl1-WT and Crtl1-KO mice after memory recall. Scale bar, 200 μm. *n* = 8 Crtl1-KO, *n* = 8 Crtl1-WT. CS = conditioned stimulus; US = unconditioned stimulus

To analyze neuronal activation in Crtl1-KO and WT immediately after memory recall, we performed immunostaining for Zif268 in a different group of conditioned Crtl1-KO and WT mice perfused immediately after presenting one block of two CS (Fig. 6A). We found that Zif268 + cells were higher in LA, BLA, CeM, CeL, and IL of Crtl1-KO than in WT mice (Fig. 6B-E). This result is in line with the behavioral data shown in Fig. 1D, 4B, and 5D, in which we found that Ctrl1-KO mice showed a consistent trend for higher freezing levels in response to the first block of two CS presented during early extinction. Thus, both behavioral and molecular data suggest in Crtl1-KO mice a more flexible circuit compared to WT, characterized by not only stronger memory recall but also faster and more persistent fear extinction (Fig. 5D-H).

## Discussion

Our results show that the removal of the Crtl1 protein that selectively disrupts the aggregation of CSPGs in PNNs is sufficient to promote an accelerated and persistent fear memory erasure after administration of an extinction protocol. Indeed, during early extinction, Crtl1-KO mice showed reduced freezing levels as soon as the 3rd block of 2 CS while Crtl1-WT mice started showing freezing reduction at the end of the second day of extinction. These results were confirmed also testing mutant and WT mice in a virtual fear conditioning protocol, and using pupil dynamics as a physiological readout of fear learning and extinction. In humans, fear conditioning is often probed by measuring autonomic responses [35], such as skin conductance responses or startle responses [23, 36]. In recent years, these physiological measures have been complemented by pupil dilations [22–24]. Pupillometry offers a promising, complementary method for the quantification of the conditioned response. Indeed, pupil size assessment is not aversive, and it can be easily combined with other measurements and provides information about the activity of the autonomic nervous system [25] and locus coeruleus noradrenergic system [37], which plays an important role in modulating fear responses and extinction [38]. Coherent with the behavioral results, we found in Crtl1-KO mice an accelerated reduction in the pupillary response to CS with respect to Crtl1-WT mice as soon as the first day of extinction.

We have also demonstrated that the condensation of CSPGs in PNNs triggered by neuronal production of the Crtl1 protein is needed for the transition from a conditioned fear memory that can be erased by extinction to a conditioned fear memory which is not. In order to assess if the observed acceleration in fear extinction translates in lower fear response at later stages, we tested freezing levels of Crtl1-KO and Crtl1-WT mice in the extinction context (spontaneous recovery) and in the training context (fear renewal) 7 and 42 days after late extinction. Interestingly, for both time points, we found a reduced spontaneous recovery and fear renewal in Crtl1-KO mice with respect to Crtl1-WT. Since early and late extinction were performed immediately after learning, the observed accelerated fear extinction pattern and the reduced freezing shown during spontaneous recovery and fear renewal may be the effect of an impairment in memory consolidation and a manifestation of oblivion. To rule out this possibility, we tested another group of Crtl1-KO and Crtl1-WT mice with a delay of 9 days between the end of the learning phase and the beginning of early extinction. We found that Crtl1-KO mice recapitulated the previous results, revealing that mutant mice do not show an impairment in memory consolidation and retrieval with respect to Crtl1-WT mice.

Our behavioral and physiological data are in accordance with the results of Gogolla et al. [7], in which the injection of the chondroitinase ABC enzyme in the BLA is able to induce the acquired fear memories susceptible to erasure. In adult animals, the organization of CSPGs in PNNs is a key event in the control of central nervous system plasticity and in the closure of critical periods in many brain regions [7, 39, 40]. In the visual cortex, it has been shown that the developmental condensation of CSPGs in PNNs, rather than their sheer presence, play a crucial role in protecting adult visual cortical circuits from being modified by experience. The response of visual cortical circuits to monocular deprivation, which can be reinstated in the adult visual cortex by enzymatic removal of CSPGs [39], is also present in mice lacking Crtl1, which have attenuated PNNs but unchanged overall levels of CSPGs [20]. Overall, our data suggest the possibility that common general mechanisms of critical period closure exist in different brain circuits.

The exact mechanism of action of PNNs in plasticity is only partially known [41]. Recently, superresolution microscopy provided a detailed description of the tight relationship between the PNN and the synaptic microstructure [42]. It would be of great interest to analyze if these high-resolution features are affected by Crtl1 mutation and if they correlate with memory changes.

The extinction of conditioned fear memories in adults relies on a network of structures, such as the amygdala, the vmPFC, and the hippocampus [9, 43]. In particular, when the CS is present, the LA excite glutamatergic neurons in the BLA and GABAergic neurons in the lateral and medial intercalated cells (ITCs) [44] which separates the BLA from the central nucleus (CeA) [45, 46]. LA and BLA project dense glutamatergic synapses onto CeA, with the LA projecting only to its lateral sector (CeL) [47], and the BLA projecting to both the lateral and the medial (CeM) sectors [48–50]. In addition, convergent evidence suggests that the vmPFC, and in particular the IL [51], is necessary for the retention and recall of extinction [52–55]. To elucidate the functional activation of amygdala and IL cortex in Crtl1-KO mice, we replicated our previous extinction experiment trying to isolate the effect of the association between the CS and the US. We compared the neuronal activation of a group of conditioned Crtl1-KO and Crtl1-WT mice with a group of pseudo-conditioned Crtl1-KO and Crtl1-WT mice, in which the CS and US were unpaired. In particular, we performed immunohistochemistry for the immediate early gene Zif268 after early extinction. Zif268 is a member of the zinc finger transcription factor family. It regulates the expression of various late-response genes involved in different neuronal processes, including synaptic plasticity [29]. The role of Zif268 and other immediate early genes, such as c-fos and Arc, in learning and memory has been well described [56]. Zif268 expression is known to increase shortly after fear conditioning [57], suggesting its importance in fear memory formation [58]. Studies in mutant mice have also shown that overexpression of Zif268 enhanced resistance to extinction of aversive memories [59], while failure to induce Zif268 allowed spontaneous recovery [59, 60]. Here we provide further evidence of Zif268’s role as a marker of activity and plasticity changes in the amygdala and IL cortex after fear extinction. Indeed, the density of Zif268+ cells revealed a significant reduction in BLA, LA, and CeM activation of Crtl1-KO mice with respect to Crtl1-WT mice. Notably, Crtl1-KO mice activation was not significantly different from pseudo-conditioned mice. The reduced activation of CeM, as the output of the amygdala, is in line with the reduced behavioral freezing shown by Crtl1-KO mice. Remarkably, the reduced activation of LA is coherent with the possibility that the behavioral reduction of freezing shown in Crtl1-KO mice may be implemented as early as in LA. Regarding IL, we did not find significant differences between Crtl1-KO and Crtl1-WT mice; however, Zif268+ cell density of Crtl1-KO mice showed a reduced variability and the levels are at the significance threshold. Since it has been shown that optogenetic activation of IL for 30 s is able to accelerate and reduce freezing levels [34], it could be hypothesized that in Crtl1-KO mice IL may be precociously (as soon as the first block of 2 CS) and increasingly recruited, with respect to Crtl1-WT mice, contributing to the accelerated reduction of fear. During the fifth block of 2 CS, IL activation would be decreased (or comparable to the Crtl1-WT mice), since fear reduction has already been achieved. We also evaluated the activation of amygdala and IL neurons in Crtl1-KO and WT mice immediately after conditioning (memory recall). Our results revealed a higher number of Zif268+ neurons in Crtl1-KO mice compared to WT mice in both the amygdala nuclei and IL cortex. This observation aligns with previous research demonstrating enhanced memory following degradation of PNNs [61, 62]. It is plausible that the more flexible circuits present in Crtl1-KO mice not only lead to stronger fear conditioning but also facilitate faster and longer-lasting extinction.

Taken together, our behavioral and molecular findings suggest that the aggregation of CSPGs into PNNs is a key factor in regulating the boundaries of the critical period for fear memories. Moreover, we suggest a possible activation pathway responsible for the accelerated reduced behavioral freezing shown by Crtl1-KO mice after extinction. We also propose pupillometry as a complementary physiological readout of fear learnings, which can be easily coupled with other physiological measures that require head-fixation procedures.

## Materials and Methods

### Animals

For this study, we used adult mice (P75-P120) lacking the Crtl1/Hapln1 gene in the CNS, but not cartilage, which leads to attenuated PNNs in the adult brain (Crlt1-KO) [63] and their wild-type littermates control (Crtl1-WT) mice. In our experiments, both male and female mice were utilized as study subjects. To control for the potential influence of sex on our results, we conducted a thorough evaluation of any sex-specific effects across all experiments. Our findings indicated that there were no significant differences observed that could be attributed solely to sex (data not shown). Because the Crtl1 product is essential for cartilage, Crtl1 was disrupted globally (Crtl1^−/−^) and then reintroduced under the control of the type II collagen-cartilage-specific promoter by crossbreeding with a second transgenic mouse line (Crtl1-Tg), as better described in Czipri et al. The resulting Crtl1^−/−^/Crtl1-Tg^+/+^ mice were on a BALB/C background; thus, for this study, they were backcrossed into a C57BL/6 J background for seven generations as described in Carulli et al. [20]. Animals’ genotypes were identified through PCR on tail tissue (P10-P12), with primers for wild-type Crtl1, disrupted Crtl1, and Crtl1 transgene expressed in cartilage [20]. Mice were housed in groups, from two to five animals per cage (60 cm × 40 cm × 20 cm), and maintained in rooms at 22 °C with a standard 12-h light–dark cycle. Food (standard diet, 4RF25 GLP Certificate, Mucedola) and water were available ad libitum and changed weekly. Open-top cages with wooden dust-free bedding were used. All the experiments were carried out according to the directives of the European Community Council (2011/63/EU) and approved by the Italian Ministry of Health. All necessary efforts were made to minimize both stress and the number of animals used. One week before the start of behavioral experiments, all mice were handled daily by the experimenter for 5 min using an open hand approach to minimize anxiety and stress response induced by experimenter manipulation [64].

### Fear Conditioning

Mice were subjected to an auditory fear conditioning and extinction procedure using a custom-made PVC fear conditioning setup (50 cm × 15 cm × 21 cm). During the test days, the mice were transported in their home cages to a room adjacent to the testing room and left for 2 h before behavioral testing. We used two different contexts: In context A, the walls and the floor were completely black; in context B, the walls had white vertical plastic strips 2.5 cm long, 0.5 cm wide, and 18 cm deep, interposed every 2.5 cm, and white floor. Both chambers were covered with transparent plexiglass lids with a loudspeaker in its center point. The shock grid on the floor was made of stainless steel. Only the grid of context A was electrified by a shock generator (World Precision Instruments, Sarasota, FL) guided by an automated program for CS and US parameter control and footshock delivery. Mice behavior was recorded by a camera controlled by the EthoVision XT 8 software (Noldus Information Technology, The Netherlands). The two chambers were cleaned with 70% ethanol before and after each animal. After each session, mice were housed separately until the end of the test to avoid possible observational fear learning.

### Conditioning and Extinctions

During the habituation day (day 0), mice were placed in context A for 3 min. On day 1 (conditioning), mice were placed again in the context A and conditioned using 5 pairings of the CS (total CS duration 10 s, 7.5 kHz, 80 dB) co-terminating with a US (1 s footshock, 0.6 mA, inter-trial interval: 20 s). On day 2 (early extinction) and day 3 (late extinction), conditioned mice were subjected to the extinction training in context B during which they received 12 unreinforced presentations of the CS on each day. We also tested fear spontaneous recovery and context-dependent fear renewal 7 and 42 days after late extinction using 4 unreinforced presentations of the CS in context B and A respectively. Fear memory retention was tested by submitting an additional group of mice to extinction training 9 days after conditioning, during which they received 10 unreinforced presentations of the CS.

### Amygdala and Infralimbic Cortex Activation After Early Extinction

To assess amygdala and IL activation, after early extinction, a separate group of conditioned Crtl1-KO and Crtl1-WT mice was used. Mice were subjected to the early extinction protocol during which they received 10 unreinforced presentations of the CS. At the end of the extinction training, mice were sacrificed for immunohistochemistry analysis. To isolate the specific effect of associative learning on amygdala and IL activation, we used pseudo-conditioned mice. For pseudoconditioning, on the day of conditioning, mice received the same number of CS as conditioned mice, administered at 1 s interstimulus intervals in context A, without US. Then, mice were placed back in their home cage. After 45 min, the animals were placed again in the context A where they immediately received 5 US at 1 s intervals (1 s footshock, 0.6 mA). This procedure has been designed to make it difficult for animals to associate the US with the CS and the context [30]. On day 2, pseudo-conditioned mice were subjected to the same early extinction protocol of conditioned mice and were then sacrificed for immunohistochemistry.

### Analysis of Freezing Behavior

Recorded videos were manually scored for freezing behavior by two separate experimenters blind to genotype and experimental conditions. Mice were considered to be freezing if no movement was detected for 2 s (defined as the complete absence of movement except for respiratory movements). For all fear conditioning and extinction paradigms, cue evoked freezing behavior was analyzed by calculating the percentage time an animal spent freezing during a given CS presentation, and averages were calculated by pooling freezing across 2 CS presentations if not indicated otherwise.

### Virtual Fear Conditioning

During the virtual fear conditioning, mice were head-fixed. We employed a custom-made apparatus equipped with a 3D printed circular treadmill (diameter: 18 cm) as described in Mazziotti et al. [28]. During each head-fixation session, a curved monitor (24 inches Samsung, CF390) was placed in front of the animal (at a distance of 13 cm). We designed two different virtual environments composed of a γ-linearized procedural virtual corridors written in C# and Unity. The two environments presented sine-wave gratings (context A) or plaid-wave gratings (context B) at different orientations (wall at 0°; floor at 90°), and spatial frequencies (from 0.06 to 0.1 cycles/°). The apparatus was cleaned before and after each animal with 70% ethanol or 1% acetic acid for context A and context B respectively, since the mice may associate the smell with the context. The position of the animal in the virtual corridor was updated using an optical computer mouse, positioned below the circular treadmill, that interfaced with the virtual environment software. As a CS, we used a visual stimulus consisting of a square wave grating patch of 55° (in width and height) of visual space in the binocular portion of the visual field. The grating parameters were as follows: luminance, 8.5 cd/m2; orientation, 0°; contrast, 90%; spatial frequency, 0.1 cycles/°; and drifting, 0.5 cycle/s. A custom-made electrode connected to a shock generator (World Precision Instruments, Sarasota, FL) and controlled by the virtual environment software was positioned on the mouse tail for tail shock delivery (US).

For fear conditioning in the virtual environment, mice were introduced gradually to head fixation and to the tail electrode for 5 days (habituation). During the habituation, we performed two sessions of head fixation in which mice were exposed to the two contexts. Each session consisted of 2 min of a uniform gray (luminance, 8.5 cd/m2), to assess the pupil diameter in baseline, and 10 min of the isoluminant virtual environment. After habituation, mice underwent a 4-day fear conditioning training and extinction protocol. On day 1, a group of mice were exposed to context A and a second group to context B and conditioned using 5 pairings of the CS (total CS duration 20 s) co-terminating with a US (2 s tail shock, 0.6 mA, inter-trial interval: 120 s). Fear memory was tested on day 2 in a recall session, presenting 5 unreinforced presentations of the CS in the context opposite of the conditioning one. On day 3 (early extinction) and day 4 (late extinction), conditioned mice were subjected to the extinction training again in the context opposite of the conditioning one during which they received on each day 10 unreinforced presentations of the CS. To test the efficacy of the virtual fear conditioning protocol, we compared the responses of a group of conditioned C57BL/6 J WT mice (shock) with a group of mice that underwent the same conditioning protocol without receiving the tail shock (sham). After each session, mice were housed separately until the end of the test to avoid possible observational fear learning.

### Pupillometry

During the virtual fear conditioning paradigm, we analyzed pupil diameter as a physiological readout of fear response. To record the pupil, we used a USB camera (oCam-5CRO-U, Withrobot Lens: M12 25 mm) connected to a Jetson AGX Xavier Developer Kit (NVIDIA) running a custom Python3 script (30 fps). Real-time pupillometry was performed using MEYE, a convolutional neural network that performs online pupillometry in mice and humans. Pupillometry data has been analyzed using Python 3. All tracks were loaded, and blink removal was applied using the blink detector embedded in MEYE. Blink epochs were filled using linear interpolation and median filtering (0.5 s). The *z*-score was obtained for each trial using the formula *z* = (*x* − *x*¯_baseline_)/*s*_baseline_, where *x*¯_baseline_ and *s*_baseline_ are respectively the average and the SD of the baseline.

### Pupillary Light Reflex

For the evaluation of the PLR, we presented 10 s of a white screen (luminance: 30 cd/m2) repeated ten times, interspersed with 50 s of a uniform gray (luminance, 8.5 cd/m2). Each session started with 2 min of a uniform gray (luminance, 8.5 cd/m2) for pupil adaptation. We evaluated the pupil constriction latency (the time needed to reach the minimal pupil size during pupil constriction), the pupil constriction amplitude (maximal relative change in pupil area during constriction), the pupil re-dilation latency (the time needed to reach the maximal pupil size during pupil re-dilation), and the pupil re-dilation amplitude (maximal relative change in pupil area to recover the constriction).

### Surgery

Mice were deeply anesthetized using isoflurane (3% induction, 1.5% maintenance), placed on a stereotaxic frame and head-fixed using ear bars. Prilocaine was used as a local anesthetic for the acoustic meatus. Body temperature was maintained at 37° using a heating pad, monitored by a rectal probe. The eyes were treated with a dexamethasone-based ophthalmic ointment (Tobradex, Alcon Novartis) to prevent cataract formation and keep the cornea moist. Respiration rate and response to toe pinch were checked periodically to maintain an optimal level of anesthesia. A subcutaneous injection of Lidocaine (2%) was performed prior to scalp removal. The skull surface was carefully cleaned and dried, and a thin layer of cyanoacrylate was poured over the exposed skull to attach a custom-made head post that was composed of a 3D printed base equipped with a glued set screw (12 mm long, M4 thread, Thorlabs: SS4MS12). The implant was secured to the skull using cyanoacrylate and UV curing dental cement (Fill Dent, Bludental). At the end of the surgical procedure, the mice recovered in a heated cage. After 1 h, mice were returned to their home cage. Paracetamol was used in the water as antalgic therapy for 3 days. We waited for 7 days before performing head-fixed pupillometry to provide sufficient time for the animals to recover.

### Immunohistochemistry

For immunofluorescence labeling, following the early extinction protocol, conditioned and pseudo-conditioned mice were transcardially perfused with 4% paraformaldehyde in phosphate buffer 1 h after behavioral testing, when Zif268 peaks in its expression [65]. Brains were removed and post-fixed for 24 h at 4 °C in 4% paraformaldehyde and then cryoprotected for 72 h at 4 °C in 20% sucrose and 0.05% sodium azide in PBS, pH 7.4. Brains were then snap-frozen in 2-methylbutane and cryosectioned in OCT using a cryostat (Leica Biosystems, CM 3050S) to obtain 40-μm-thick sections collected in PBS. Free-floating sections were incubated for 2 h at room temperature (RT) in a blocking solution composed of 10% bovine serum albumin (BSA), 0.5% Triton X-100, in PBS. For Zif268 staining, sections were incubated overnight at 4 °C in a solution composed of 10% BSA, 0.3% Triton X-100, and 1:500 rabbit polyclonal anti-Zif268 primary antibody (SantaCruz), in PBS. Sections were then washed for 3 times, 10 min each time. Primary antibody was revealed by incubating sections for 2 h at RT in a solution composed of 1% BSA, 0.1% Triton X-100, and 1:400 goat anti-rabbit AlexaFluor 488 secondary antibody (Invitrogen), in PBS. Sections were then washed for 3 times, 10 min each time and mounted on glass slides and covered with VectaShield mounting medium (Vector). For the quantification of PNNs, mice were transcardially perfused and the 40-µm coronal sections were cut on a freezing microtome (Leica). Slices were incubated for 2 h RT in a blocking solution composed of 3% BSA in PBS. Then, slices were incubated overnight at 4 °C with a solution containing biotinylated Wisteria floribunda Lectin (WFA, B-1355–2, Vector Laboratories, 1:200) and 3% BSA in PBS. On the following day, sections were washed for 3 times in PBS (10 min each) and incubated with a solution of red fluorescent streptavidin (Streptavidin, Alexa Fluor™ 555 con488 jugate, S21381, Thermo Fisher, 1:400) and 3% BSA in PBS for 2 h at RT, and washed again 3 times in PBS and subsequently mounted on glass slides and covered with VectaShield mounting medium. Sections were acquired at 16X using a confocal laser scanning microscope (Leica Biosystems, BM 6000) and digitized with Leica confocal software. From 3 to 5 sections were acquired for each amygdala nucleus and IL for each animal. Zif268-positive cells and PNNs were manually counted using the MetaMorph software and ImageJ software by two separate experimenters blind to genotype and experimental conditions.

### Statistical Analysis

All statistical analyses were performed using GraphPad Prism 7 and Python custom scripts (Pupillometry). Parametric *t*-test, analysis of variance (ANOVA), and repeated measure-ANOVA (RM-ANOVA) were used. ANOVA was followed by appropriate post hoc tests. Significance was set at *P* < 0.05 for all tests. Error bars represent s.e.m. in all figures.

## Supplementary Information

Below is the link to the electronic supplementary material.

Supplementary file1 (DOCX 3.34 MB)

## Data Availability

The datasets generated during and/or analyzed during the current study are available from the corresponding author on reasonable request.
