# Supplementary material for: Selective Disruption of Perineuronal Nets in Mice Lacking Crtl1 is Sufficient to Make Fear Memories Susceptible to Erasure
Source: Mol Neurobiol. 2023 Apr 6;60(7):4105–19. doi: 10.1007/s12035-023-03314-x (PMC10224842; doi:10.1007/s12035-023-03314-x)
Supplement: Supplementary file 1 — Supplementary file1 (DOCX 3.34 MB) [file 12035_2023_3314_MOESM1_ESM.docx]

**Supplementary Material**

**Selective disruption of perineuronal nets in mice lacking Crtl1**

**is sufficient to make fear memories susceptible to erasure**

**Journal name: Molecular Neurobiology**

Andrea Poli^1^*^$^*, Aurelia Viglione^1^*^$^*, Raffaele Mazziotti^2^, Valentino Totaro^1^, Silvia Morea^2^, Riccardo Melani^3^, Davide Silingardi^4^, Elena Putignano^2^, Nicoletta Berardi^2,4^ and Tommaso Pizzorusso^1,2,*^

*1. BIO@SNS Lab, Scuola Normale Superiore via G. Moruzzi 1, 56124 Pisa, Italy*

*2. Institute of Neuroscience, National Research Council, Via Moruzzi, 1 56124 Pisa, Italy*

*3. Neuroscience Institute, New York University Grossman School of Medicine, New York, NY 10016, USA*

*4. Department of Neuroscience, Psychology, Drug Research, and Child Health NEUROFARBA, University of Florence, 50134 Florence, Italy*

** Corresponding author: Tommaso Pizzorusso, Istituto Neuroscienze CNR and BIO@SNS lab, Scuola Normale Superiore via G. Moruzzi 1, 56124 Pisa, Italy, tel +390503153167, Fax +390503153220, e-mail:* [*tommaso.pizzorusso@in.cnr.it*](mailto:tommaso.pizzorusso@in.cnr.it)

*$ These authors contributed equally to this work*


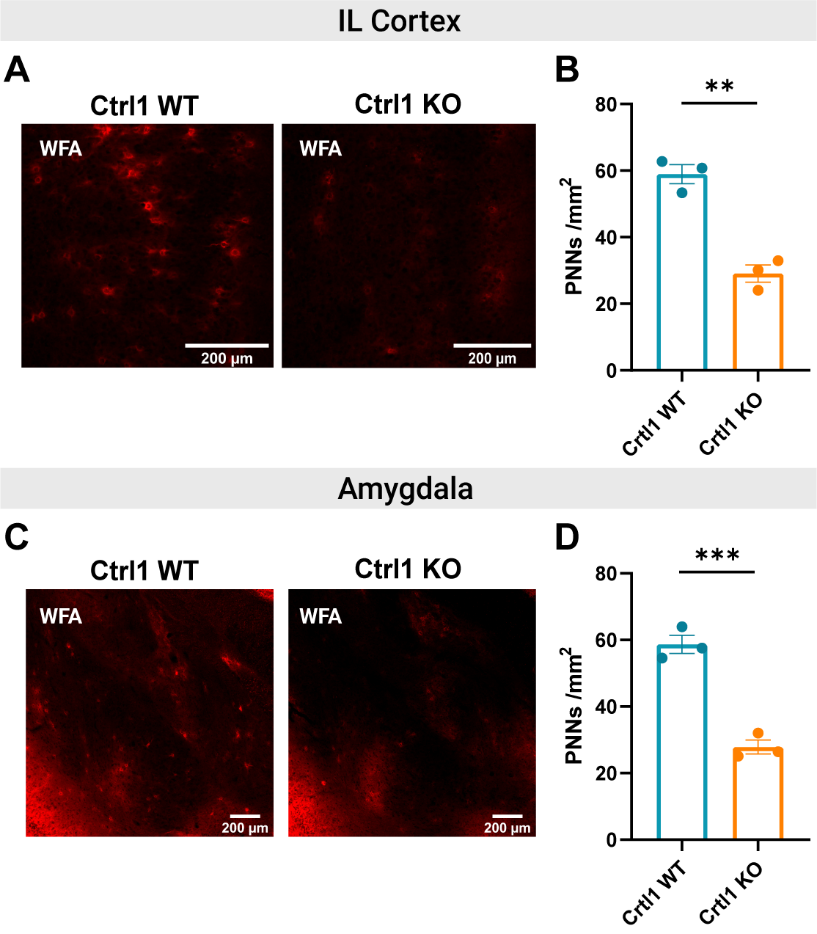


**Figure S1:** **PNNs number in the Infralimbic cortex (IL) and amygdala of Crtl1-KO and WT mice. [A]** Representative images of WFA staining in the IL cortex of Crtl1-KO and WT mice. **[B]** Number of PNNs in the IL cortex. Crtl1-KO mice present a lower number of aggregated PNNs compared to Crtl1-WT mice (Unpaired T-test p=0.0015). **[C]** Representative images of WFA staining in the amygdala of Crtl1-KO and WT mice (Unpaired T-test p=0.0015). **[D]** Number of PNNs in the IL cortex. Crtl1-KO mice present a lower number of aggregated PNNs compared to Crtl1-WT mice (Unpaired T-test p=0.0009). n = 3 Crtl1-KO, n = 3 Crtl1-WT.


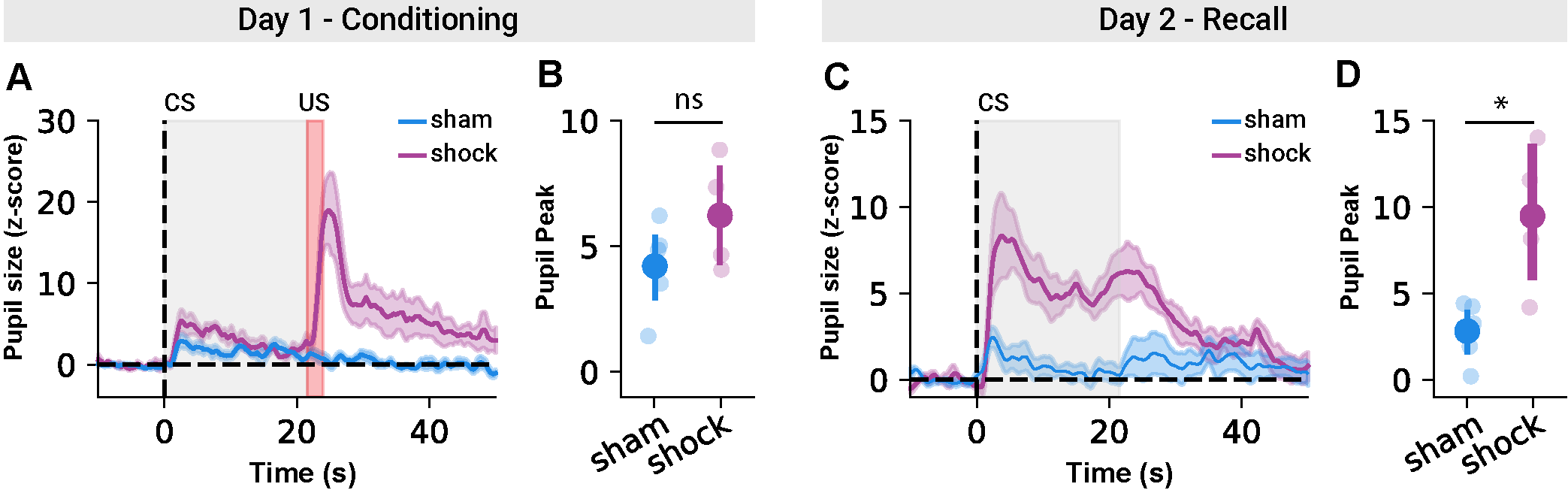


**Figure S2:** **Pupillary responses during the virtual fear conditioning in shock and sham wild-type mice. [A]** Average fluctuation of pupil size during the presentation of 5 CS (total CS duration 20 s) co-terminating (shock animals) or not (sham animals) with a US (2 s tail shock, 0.6 mA.). Gray shaded area represents the presentation of the CS and vertical dashed lines represent the onset of the CS. Red shaded area represents the presentation of the US. **[B]** Pupil peaks during the presentation of the 5 CS. We found no significant differences between groups but a trend in the pupillary response to CS in shock mice (Unpaired T-test p=0.352). Gray shaded area represents the presentation of the CS and vertical dashed lines represent the onset of the CS. **[C]** Average fluctuation of pupil size during the presentation of 5 CS the day after conditioning (Recall) in shock and sham mice. **[D]** Pupil peaks during the presentation of the 5 CS. We found a significant differences between groups. In particular shock mice showed an higher pupillary response to CS compared to sham mice (Unpaired T-test p=0.0381). n = 6 sham, n = 4 shock. CS= Conditioned Stimulus; US = Unconditioned Stimulus; ns= not significant.


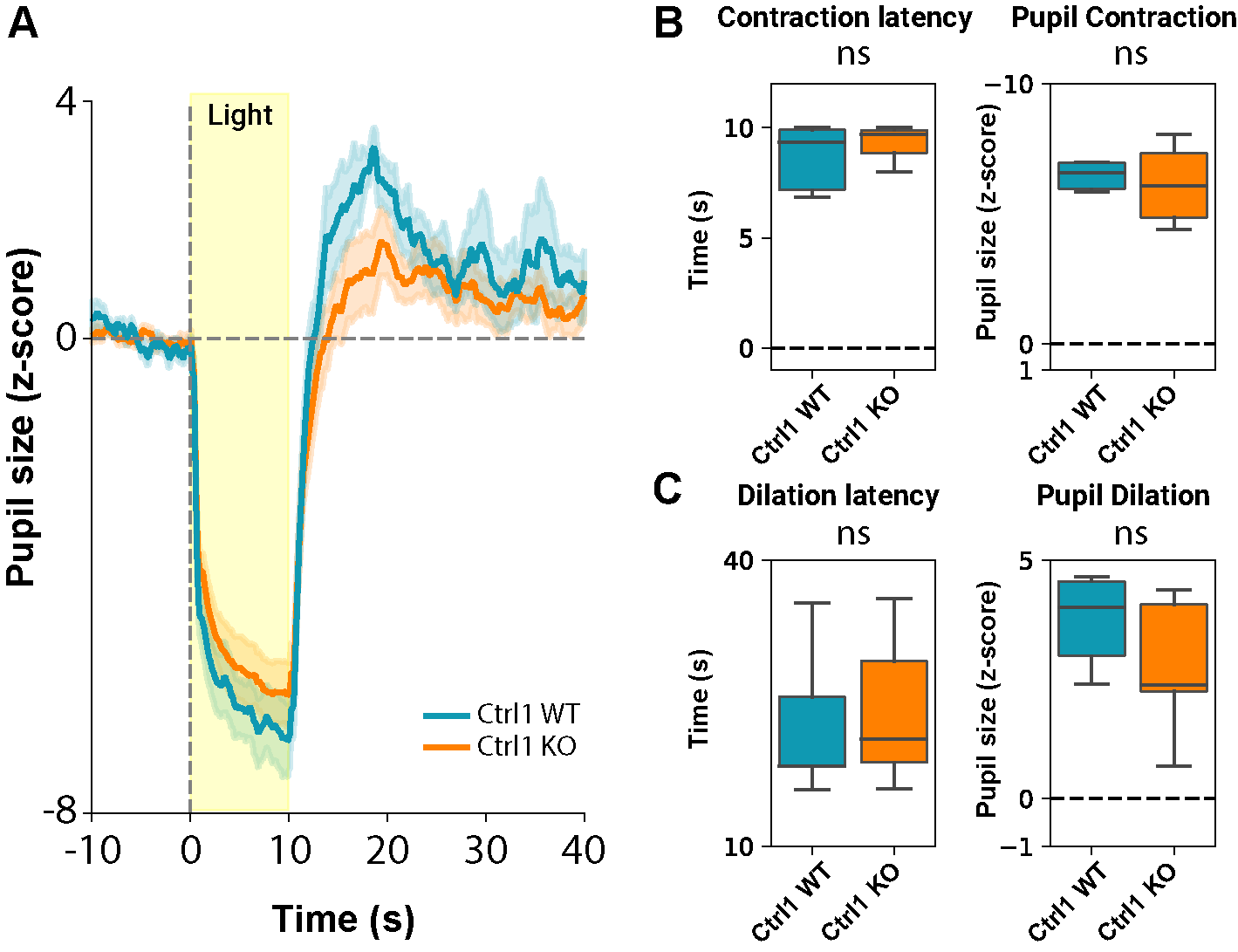


**Figure S3: Pupillary Light Reflex in Ctrl1-WT and Ctrl1-KO mice. [A]** Average fluctuation of pupil size during the presentation of the light flashes in Ctrl1-WT and Ctrl1-KO mice. Shaded area represents the presentation of the high luminance stimulus. Vertical dashed lines represent the onset of the visual stimulus. **[B]** On the left, the average contraction latency during the presentation of the light flashes. On the right, the average pupil size during contraction. For both the measures we found no significant differences between genotypes (contraction latency: Unpaired T-test p=0.404, pupil contraction: Unpaired T-test p=0.359). **[C]** On the left, the average re-dilation latency at the end of the light flashes. On the right, the average pupil size during re-dilation. For both the measures we found no significant differences between genotypes (contraction latency: Unpaired T-test p=0.759, pupil contraction: Unpaired T-test p=0.177). n = 10 Crtl1-KO, n = 11 Crtl1-WT. ns= not significant.

**
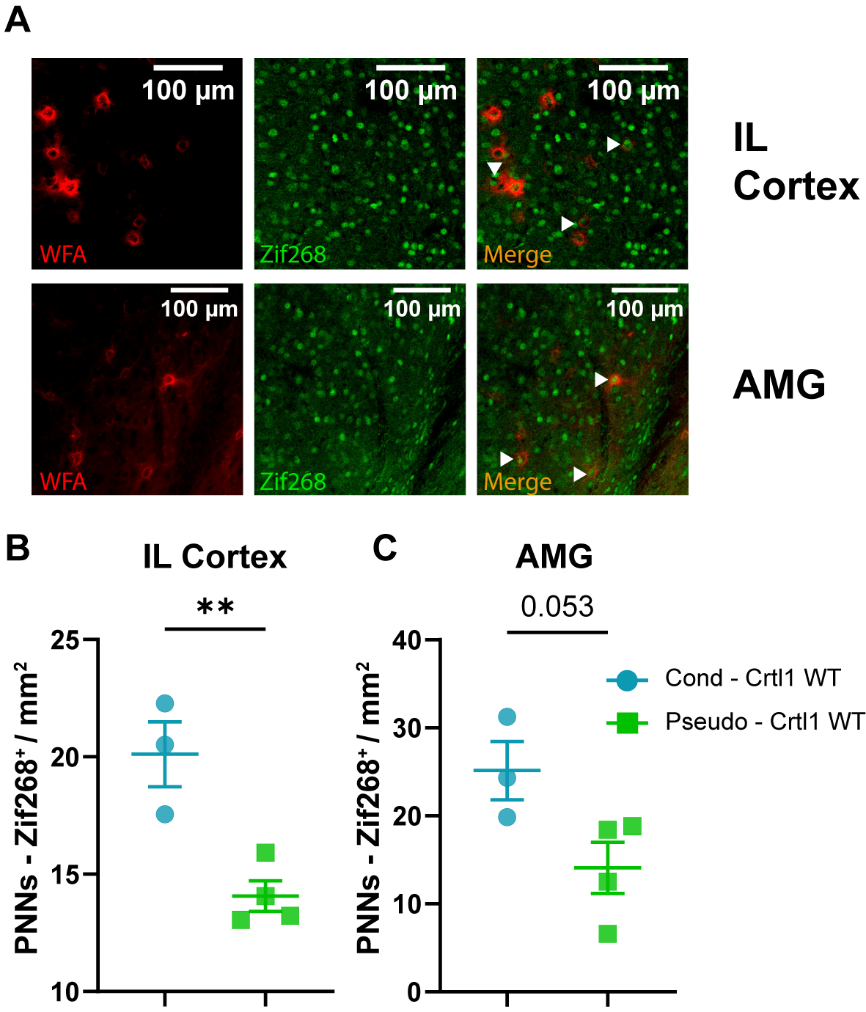
**

**Figure S4: Colocalization between PNNs and Zif268 in the infralimbic cortex and amygdala of conditioned and pseudo-conditioned Crtl1-WT mice. [A]** Double-immunofluorescence staining showing colocalization between WFA (red) and Zif268 (green) in the infralimbic cortex (IL) and amygdala (AMG) of conditioned and pseudo-conditioned Crtl1-WT mice, after early extinction. **[B]**  The number of PNNs surrounding a Zif268+ cell in the IL cortex is significantly higher in conditioned Crtl1-WT mice compared to pseudo-conditioned Crtl1-WT (Unpaired T-test, p= 0.0074). **[C]**  The number of PNNs surrounding a Zif268+ cell in the amygdala of conditioned Crtl1-WT mice is higher, albeit non statistical significant, compared to pseudo-conditioned Crtl1-WT (Unpaired T-test, p= 0.053)**.**  n = 3 Cond-Crtl1-WT **,** n = 4 Pseudo-Crtl1-WT. Scale bar, 100 μm.


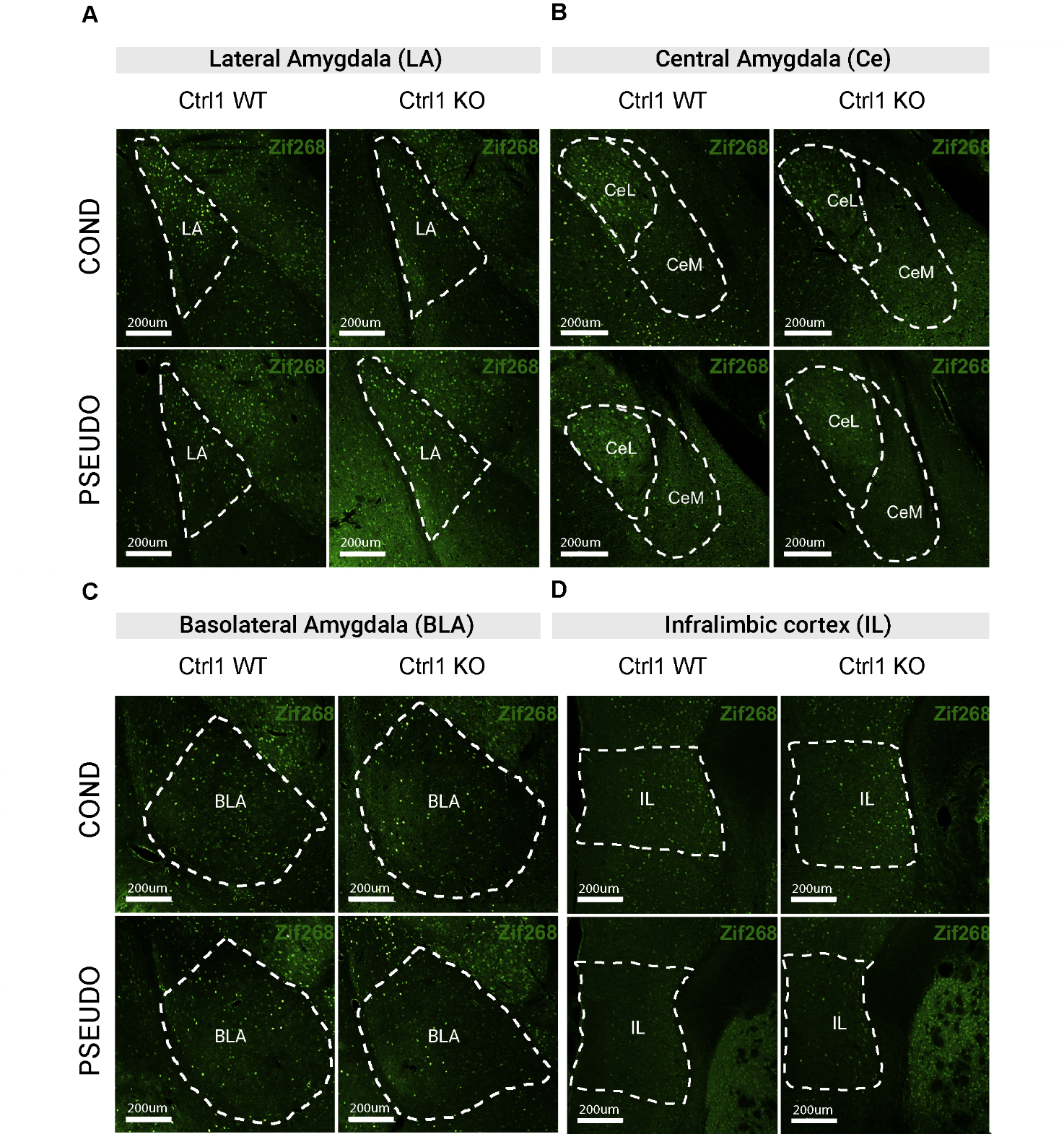


**Figure S5: Zif268 expression in amygdala and infralimbic cortex after extinction.** Representative images of Zif268-positive cells in **[A]** Lateral Amygdala, **[B]** Central Amygdala, Medial (CeM) and Lateral sector (CeL), **[C]** Basolateral Amygdala and **[C]** Infralimbic Cortex (IL) of conditioned and pseudo-conditioned Crtl1-WT and Crtl1-KO mice after the fifth block of 2 CS during early extinction. Extended version of the images in Figure 5 F and H.
